# Prevalence and determinants of Hyperpolypharmacy in adults with heart failure: an observational study from the National Health and Nutrition Examination Survey (NHANES)

**DOI:** 10.1186/s12872-019-1058-7

**Published:** 2019-04-01

**Authors:** Peter J. Kennel, Jerard Kneifati-Hayek, Joanna Bryan, Samprit Banerjee, Irina Sobol, Mark S. Lachs, Monika M. Safford, Parag Goyal

**Affiliations:** 1 Department of Medicine, Weill Cornell Medicine, New York, NY USA; 2 Division of General Internal Medicine/Department of Medicine, Weill Cornell Medicine, 525 East 68th Street, F-2010, New York, NY 10021 USA; 3 Department of Healthcare Policy & Research, Weill Cornell Medicine, New York, NY USA; 4 Division of Cardiology/Department of Medicine, Weill Cornell Medicine, New York, NY USA; 5 Division of Geriatrics/Department of Medicine, Weill Cornell Medical College, New York, NY USA

**Keywords:** Polypharmacy, Heart failure, Healthcare disparity

## Abstract

**Background:**

While an expanding armamentarium of pharmacologic therapies has contributed to improved outcomes among adults with heart failure (HF) over the past two decades, this has also been accompanied by an increase in the number of medications taken by adults with HF. The use of at least 10 medications, defined as hyperpolypharmacy, is particularly notable given its association with adverse outcomes. We aimed to assess the prevalence and identify determinants of hyperpolypharmacy among adults with HF.

**Methods:**

We studied adults aged ≥50 years with self-reported HF from the National Health And Nutrition Examination Survey (NHANES) in 2003–2014. We calculated weighted means and percentages to describe patient characteristics. We conducted a multivariable Poisson regression analysis to identify factors independently associated with hyperpolypharmacy; we adjusted for survey sampling, socio-demographics, comorbidity, geriatric conditions, and health care utilization. We examined 947 participants, representing 4.6 million adults with HF.

**Results:**

The prevalence of hyperpolypharmacy was 26%. In a multivariable regression analysis, comorbidity count, ≥10 ambulatory contacts, and ≥ 3 hospitalizations were independently associated with hyperpolypharmacy. Interestingly, functional impairment and cognitive impairment were not independently associated with hyperpolypharmacy; while low annual household income and low educational status were each associated with an almost 2-fold increase in hyperpolypharmacy.

**Conclusion:**

Hyperpolypharmacy is a common condition among adults with HF. We additionally found that low household income and low educational status are independently associated with hyperpolypharmacy, suggesting that non-medical factors may be contributing to this potentially harmful condition.

## Background

While an expanding armamentarium of pharmacologic therapies has contributed to improved outcomes among adults with heart failure (HF) over the past two decades, [1, 2] this has also been accompanied by an increase in the number of medications taken by adults with HF. [3] Indeed, in addition to the many medications specifically indicated for HF, adults with HF also frequently take medications for other common cardiovascular conditions like coronary artery disease and atrial fibrillation, as well as for non-cardiovascular conditions like lung disease and musculoskeletal disease. [4, 5] Consequently, the number of medications taken by some adults with HF could easily surpass 10. [6]

The use of at least 10 medications is particularly notable, representing a condition described in the Geriatrics and Pharmacology literature as hyperpolypharmacy. [7, 8] Hyperpolypharmacy represents an extreme version of polypharmacy—the condition of taking a high number of medications [9]—and is associated with a number of adverse outcomes including disability, [10] hospitalizations, [11] and mortality. [12] Despite the high burden of medication use among adults with HF, the concept of hyperpolypharmacy may be overlooked. This is potentially problematic, as adults with HF represent a subgroup at particularly high risk for medication-related adverse outcomes due to alterations in pharmacokinetics and pharmacodynamics, [13] changes in cardiovascular structure and function, [14] and the coexistence of geriatric conditions like frailty [15] and cognitive impairment. [16] Accordingly, we sought to examine a nationally-representative cohort of adults with HF using the National Health And Nutrition Examination Survey (NHANES) data to better understand the prevalence of hyperpolypharmacy and identify its determinants among this vulnerable population.

## Methods

To conduct this cross-sectional population-based study of survey data, we used publically available data from National Health And Nutrition Examination Survey (NHANES). NHANES is a cross-sectional survey-based study with a probability-based complex, stratified, multistage design. [17] NHANES is designed to produce national estimates representative of the total non-institutionalized civilian United States population. Weighting produces estimates of the data that would be obtained if the entire cohort that fits inclusion criteria (civilian U.S. population) had been surveyed. Health information is gathered biennially from a sample of adult non-institutionalized civilians via an interview and in-home examination.

We conducted a cross-sectional observational study of NHANES survey data. We included subjects aged ≥50 years old with self-reported HF from the 2003–2014 cycles in our analysis. Self-reported HF is a reasonable approach to identifying a nationally representative cohort of patients with HF, as statistics on self-reported HF derived from NHANES are used in the annual report on heart disease and stroke by the American Heart Association [15] and self-report has previously demonstrated a high specificity for HF. [15] We excluded subjects with missing data on self-reported HF and/or medications. We examined several variables routinely collected in NHANES. This included socio-demographic variables (age, gender, race, payer status, highest education attained, household income, marital status, and living alone); comorbid conditions available during each NHANES cycle included in the study period (hypertension, diabetes, anemia, asthma, chronic bronchitis, emphysema, coronary artery disease, prior myocardial infarction, prior stroke, thyroid disease, liver disease, cancer, chronic kidney disease, dialysis, hypercholesterolemia, arthritis); smoking (never, past, or current); self-reported health compared to the prior year (same, worse, or better); geriatric conditions (self-reported memory problems as a proxy for cognitive impairment, and functional impairment as characterized by impaired activities of daily living based on “difficulty getting in and out of bed,” had “difficulty using a fork, knife, drinking from cup” or difficulty “dressing themselves”); and healthcare utilization (number of contacts with ambulatory healthcare services and emergency room visits that did not result in an overnight hospitalization, and the number of hospitalizations in the prior year).

During the in-home examination, participants were asked to report prescription medications that they had taken during the prior month, and show their medication containers to the examiner. We defined hyperpolypharmacy as the condition of taking ≥10 medications. [7, 8] We classified medications according to the Multum Lexicon Drug Database, [18] which then facilitated a broader classification scheme of three major medication groups—HF medications, other (non-HF related) cardiovascular medications, and non-cardiovascular medications.

For all analyses, we accounted for the complex survey design of NHANES. In particular, we calculated weighted percents and/or weighted means for all variables, and accounted for clustering in all analyses. We assessed significant differences between those with and without hyperpolypharmacy by performing a Pearson’s chi square test for categorical variables and a t-test for continuous variables. Since the sample size is large, a simple application of the Lyapunov or Lindeberg’s Central Limit Theorem guarantees large sample convergence of the weighted mean to a standard normal distribution, ensuring that the t-statistic would have a limiting t-distribution.

To identify factors independently associated with hyperpolypharmacy, we performed a Poisson regression analysis with robust standard errors that incorporated all candidate variables including socio-demographics, comorbidity count (simple count of all comorbid conditions present for each participant), smoking status, change in self-reported health, geriatric conditions, and health care utilization. Referent groups were chosen based on high prevalence and/or condition of low risk. To account for missing values for covariates included in the regression analysis, we performed multiple imputation using chained equations designed for complex survey data. [19] All statistical tests were two-sided, with a *p*-value < 0.05 indicating statistical significance. We performed all analyses with SAS version 9.4 and STATA version 14.

## Results

We studied 947 survey respondents, which represented 4.6 million non-institutionalized participants from the United States. Baseline characteristics are shown in Table 1. The cohort had a mean age of 70 years (±8.9), about half (49%) were women, and the majority were white (77%). Most participants were Medicare beneficiaries (66%), most had an educational level below a college degree (87%), and a substantial proportion reported an annual household income of under $20,000 (27%).

**Table 1:** Population Characteristics According to Hyperpolypharmacy

Variable	All	No HPP (*n* = 705)	HPP (*n* = 242)	*P*-value
Mean age (SD)	70.0 (8.9)	70.1 (9.1)	69.7 (8.2)	0.67
Age, %				0.26
50–74	61	60	65	
≥75	40	41	35	
Women, %	49	48	54	0.07
Race, %				0.04
White	77	76	81	
Black	13	13	13	
Other	10	12	6	
Primary Payer, %				0.30
Medicare without Medicaid	66	64	72	
Medicaid without Medicare	6	6	6	
Dual Medicare and Medicaid	7	7	8	
Other insurance	15	16	12	
Uninsured	6	7	3	
Education, %				0.01
High school or below	87	8	93	
College degree and above	13	15	7	
Household Income (%)				0.36
≥$75,000	14	16	10	
$35,000–$74,000	28	28	28	
$20,000–$34,000	27	27	28	
<$20,000	27	26	32	
Refused to answer	3	4	2	
Married, %	53	52	56	0.38
Living alone, %	26	27	24	0.42
Count of comorbidities, mean (95% CI)	4.7 (4.5–4.8)	4.3 (4.1–4.4)	5.9 (5.5–6.3)	<0.001
Smoking status, %				0.34
Never	40	40	39	
Past	43	42	48	
Current	16	18	13	
Change in self-reported health, %				0.27
Better	22	22	23	
Worse	22	21	27	
Same	55	57	50	
Cognitive impairment, %	24	23	26	0.52
Functional impairment, %	12	10	16	0.04
Number of contacts with ambulatory healthcare services, past year				<0.001
0–3	20	25	7	
4–9	41	42	38	
≥10	39	33	56	
Number of hospitalizations, past year				<0.001
<3	91	95	82	
≥3	9	5	18	
Cycle Year				0.78
2003–2004	17	18	16	
2005–2006	17	18	14	
2007–2008	16	15	16	
2009–2010	14	14	14	
2011–2012	19	19	20	
2013–2014	18	17	21	

Abbreviations: *HPP* Hyperpolypharmacy, *CI* Confidence Interval, *SD* standard deviation

The prevalence of hyperpolypharmacy was 26%. Those with hyperpolypharmacy took a mean number of 11.9 (±2.0) medications, compared to 5.5 (±2.5) in individuals without hyperpolypharmacy. The mean number of HF medications, non-HF cardiovascular medications, and non-cardiovascular medications for those with hyperpolypharmacy were each higher compared to those without hyperpolypharmacy (Table 2). This difference was most striking among the non-cardiovascular medications—individuals with hyperpolypharmacy took a mean number of 6.6 (±2.4) non-cardiovascular medications, compared to a mean of 2.3 (±1.8) in those without hyperpolypharmacy (*p* < 0.001). Non-cardiovascular medications comprised 55% of medications in individuals with hyperpolypharmacy, compared to 42% in those without hyperpolypharmacy (*p* < 0.001). HF medications comprised 24% of medications among those with hyperpolypharmacy, compared to 34% among those without hyperpolypharmacy (Table 3). Notably, individuals with hyperpolypharmacy more frequently took opioid and non-opioid analgesics, psychotropic substances such as benzodiazepines and antidepressants, anti-diabetic agents, antacids, thyroid agents, bronchodilators, genitourinary tract agents, supplements, anti-infective agents, and topical agents. (Table 2).

**Table 2:** Medication Profile According to Hyperpolypharmacy

Variable	All	No HPP	HPP	*P*-value
Prevalence of Hyperpolypharmacy	26%	–	–	–
Total Medication Count, mean (SD)	7.2 (3.7)	5.5 (2.5)	11.9 (2.0)	<0.001
Heart Failure Medications, mean (SD)	2.1 (1.3)	1.9 (1.3)	2.8 (1.2)	<0.001
Beta blockers, %	61	56	77	<0.001
ACEI or ARB, %	58	55	66	0.02
Aldosterone antagonist, %	11	9	16	0.01
Vasodilators, %	10	7	18	0.001
Diuretics, %	60	53	78	<0.001
Digoxin, %	13	12	17	0.08
Other Cardiovascular Agents, mean (SD)	1.6 (1.3)	1.3 (1.1)	2.5 (1.3)	<0.001
Lipid Lowering, %	60	53	80	<0.001
Anti-platelet agents, %	21	14	40	<0.001
Anti-coagulation agents, %	21	19	26	0.06
Anti-arrhythmic agents, %	26	23	32	0.02
Calcium-channel blockers, %	22	19	31	0.001
Anti-anginal agents, %	12	7	27	<0.001
Other anti-hypertensive agents, %	14	10	24	<0.001
Non-cardiovascular medications, mean (SD)	3.4 (2.7)	2.3 (1.8)	6.6 (2.4)	<0.001
Opioids, %	15	9	30	<0.001
Non-opioid analgesic, %	11	8	19	0.004
Benzodiazepines, %	9	6	18	<0.001
Anti-depressants, %	23	17	41	<0.001
Anti-psychotics, %	3	2	3	0.6
Anti-diabetic agents, %	34	27	55	<0.001
Antacids, %	32	23	60	<0.001
Thyroid agents, %	20	16	30	<0.001
Bronchodilators, %	15	9	29	<0.001
GU Tract agents, %	4	2	8	0.001
Minerals/vitamins, %	24	17	45	<0.001
Anti-infective agents, %	9	6	17	0.001
Anti-neoplastic agents, %	2	1	5	0.003
Topical agents, %	9	5	17	<0.001

Abbreviations: *HPP* Hyperpolypharmacy, *CI* Confidence Interval, *ACEI* Angiotensin Converting Enzyme Inhibitor, *ARB* Angiotensin II Receptor Blocker, *PPI* Proton Pump Inhibitor, *GU* Genitourinary, *SD* standard deviation

**Table 3:** Composition of Medication Regimens According to Hyperpolypharmacy

Variable	All	No HPP	HPP	*P*-value
HF medications, %	30	34	24	<0.001
Non-HF, Cardiovascular medications, %	23	24	21	<0.001
Non-cardiovascular medications, %	48	42	55	<0.001

Abbreviations: *HPP* Hyperpolypharmacy, *HF* Heart Failure

The mean number of comorbid conditions was 4.7 (±2.0) for the entire sample of individuals with HF. Those with hyperpolypharmacy had a higher comorbidity count than those without hyperpolypharmacy (Table 4). Of note, pulmonary disorders including asthma, chronic bronchitis and emphysema were each more than 2-fold more prevalent among individuals with hyperpolypharmacy compared to those without it (Table 4). Chronic ailments that frequently require multiple medications such as diabetes (*p* < 0.001), coronary artery disease (*p* < 0.001), and arthritis (*p* = 0.001) [20] were also significantly more prevalent in individuals with hyperpolypharmacy.

**Table 4:** Comorbid Conditions According to Hyperpolypharmacy

Variable	All	No HPP	HPP	*P*-value
Comorbidity Count, mean (SD)	4.7 (2.0)	4.3 (1.8)	5.9 (2.1)	<0.001
Hypertension, %	76	74	83	0.04
Hypercholesterolemia, %	66	63	73	0.03
Myocardial Infarction, %	46	43	52	0.07
Coronary Artery Disease, %	40	35	54	<0.001
Stroke, %	22	20	27	0.05
Diabetes, %	39	32	59	<0.001
Asthma, %	17	13	28	0.001
Chronic Bronchitis, %	12	9	20	0.003
Emphysema, %	13	10	22	<0.001
Cancer, %	27	27	25	0.70
Anemia, %	12	11	15	0.20
CKD, %	14	10	24	<0.001
Dialysis, %	2	2	3	0.60
Thyroid disease, %	18	15	26	0.002
Arthritis, %	63	58	76	0.001
Liver disease, %	4	4	5	0.42

Abbreviations: *HPP* Hyperpolypharmacy, *CI* Confidence Interval, *CKD* Chronic Kidney Disease, *SD* standard deviation

Table 5 shows a multivariable regression analysis of determinants for hyperpolypharmacy. Notably, comorbidity count (1.19, 95% CI [1.12–1.27], *p* < 0.001), ≥ 10 ambulatory contacts (3.01, 95% CI [1.73–5.21] *p* < 0.001) and ≥ 3 hospitalizations (1.70, 95% CI [1.27–2.30] *p* = 0.001) were strongly associated with hyperpolypharmacy. In addition, an educational level of below college (1.74, 95% CI [1.01–2.99] *p* = 0.04) and a household income below $20,000 (1.70, 95% CI [1.01–2.85] p = 0.04) were independently associated with hyperpolypharmacy. Meanwhile, advanced age, geriatric conditions including functional impairment and cognitive impairment, and declining health were not independently associated with the presence or absence of hyperpolypharmacy (Table 5).

**Table 5:** Prevalence Ratios for the Determinants of Hyperpolypharmacy

Variable	Univariate Model		Multivariable Model	
	PR (95% CI)	*P*-value	PR (95% CI)	*P*-value
Age ≥ 75	0.84 (0.62–1.14)	0.26	0.83 (0.63–1.11)	0.21
Women	1.22 (0.98–1.52)	0.07	1.15 (0.92–1.43)	0.23
Race				
White	Referent Group		Referent Group	
Black	0.96 (0.70–1.32)	0.81	0.98 (0.71–1.35)	0.91
Other	0.56 (0.34–0.93)	0.03	0.59 (0.38–0.91)	0.02
Primary Payer				
Medicare without Medicaid	Referent Group		Referent Group	
Medicaid without Medicare	1.03 (0.60–1.76)	0.91	0.76 (0.48–1.21)	0.25
Dual Medicare and Medicaid	1.03 (0.67–1.59)	0.88	0.82 (0.53–1.27)	0.37
Other insurance	0.76 (0.39–1.48)	0.42	0.85 (0.50–1.45)	0.55
Uninsured	0.40 (0.15–1.12)	0.08	0.66 (0.25–1.73)	0.40
Education, High school or below	1.95 (1.12–3.38)	0.02	1.74 (1.01–2.99)	0.04
Household Income				
≥$75,000	Referent Group		Referent Group	
$35,000–$74,000	1.40 (0.80–2.45)	0.24	1.52 (0.89–2.59)	0.12
$20,000–$34,000	1.48 (0.85–2.65)	0.17	1.42 (0.86–2.35)	0.16
<$20,000	1.65 (1.00–2.70)	0.05	1.70 (1.01–2.85)	0.04
Not Married	0.87 (0.64–1.19)	0.38	0.82 (0.56–1.21)	0.32
Lives alone	0.89 (0.66–1.19)	0.43	0.95 (0.65–1.40)	0.81
Count of comorbidities	1.28 (1.21–1.36)	<0.001	1.19 (1.12–1.27)	<0.001
Smoking status				
Never	Referent Group		Referent Group	
Past	1.13 (0.85–1.50)	0.39	0.96 (0.72–1.28)	0.80
Current	0.78 (0.42–1.46)	0.44	0.69 (0.41–1.16)	0.16
Change in self-reported health				
Better	Referent Group		Referent Group	
Same	0.86 (0.61–1.21)	0.39	1.08 (0.78–1.50)	0.64
Worse	1.14 (0.76–1.70)	0.53	1.05 (0.72–1.53)	0.80
Cognitive impairment	1.10 (0.83–1.46)	0.52	0.92 (0.68–1.24)	0.57
Functional impairment	1.46 (1.03–2.06)	0.04	1.02 (0.72–1.45)	0.90
Number of contacts with ambulatory healthcare services, past year				
0–3	Referent Group		Referent Group	
4–9	2.78 (1.52–5.08)	0.001	2.20 (1.23–3.96)	0.01
>10	4.31 (2.50–7.42)	<0.001	3.01 (1.73–5.21)	<0.001
Number of hospitalizations, past year				
<3	Referent Group		Referent Group	
≥3	2.35 (1.75–3.14)	<0.001	1.70 (1.27–2.30)	0.001
Cycle Year				
2003–2004	Referent Group		Referent Group	
2005–2006	0.88 (0.55–1.40)	0.58	0.81 (0.50–1.33)	0.41
2007–2008	1.15 (0.66–2.00)	0.63	1.16 (0.67–2.01)	0.60
2009–2010	1.12 (1.72–1.76)	0.63	1.16 (0.79–1.71)	0.44
2011–2012	1.13 (0.74–1.73)	0.57	1.16 (0.83–1.63)	0.38
2013–2014	1.27 (0.70–2.31)	0.42	1.35 (0.88–2.06)	0.16

Abbreviations: *CI* Confidence Interval, *PR* Prevalence Ratio

Within this sample, 28% had a missing value for 1 covariate, and only 4% had a missing value for more than 1 covariate. Variables with the highest percent missing included primary payer (17%), comorbidity count (13%), and income (1%). All other variables were missing < 1% in the sample. Participants who were missing primary payer information were more likely to be white compared to the rest of the sample; and those missing comorbidity count were more likely to be female compared to the rest of the sample.

## Discussion

There are two major findings from our analysis of NHANES data. First, hyperpolypharmacy was common among adults with HF. Second, non-medical factors including low income and low education were independently associated with hyperpolypharmacy.

Our study found that one out of every four ambulatory HF patients take 10 or more medications. This is concerning because a high medication burden contributes to an increased risk for adverse drug reactions, and is associated with a number of adverse outcomes including disability, [10] hospitalizations, [11] and mortality. [12] The prevalence of hyperpolypharmacy did not vary even in the presence of factors associated with an increased risk for adverse drug events—advanced age, [21, 22] cognitive impairment, [16] and functional impairment. [10] This is consistent with a recent study of HF patients, where we showed that those with functional impairment take the same number of medications as those without functional impairment when adjusting for other factors like comorbidity burden, even when factors like cognitive impairment, low self-reported health, and recurrent hospitalizations are present. [23] This raises concern not only for drug-drug interactions, but also for drug-disease interactions, as HF is a condition that can be exacerbated by several common medications. [24, 25] High medication burden has also been linked to medication non-adherence, [26] a particularly relevant issue in HF, as self-care and medication adherence are key components of chronic disease management. Consequently, while hyperpolypharmacy may be a reflection of guideline-concordant therapy for multiple co-occurring conditions (comorbidity count was independently associated with hyperpolypharmacy), it is important to consider the potential risks of a high number of medications when prescribing medications, as adverse drug events and non-adherence can undermine the efficacy and safety of HF therapy.

When examining determinants of hyperpolypharmacy, our study revealed that non-medical factors including low income and low education were independently associated with hyperpolypharmacy, even after controlling for other socio-demographic factors including receipt of Medicaid, comorbid conditions, and healthcare utilization. This was a surprising and concerning finding that warrants additional investigation. Disparities in the outcomes of individuals with HF and low socioeconomic status (SES) are well-described—individuals with low SES are at increased risk for hospitalization and death. [27, 28] This may in part relate to suboptimal care, for which low SES is a known risk factor. For example, those with low SES are less likely to undergo an evaluation of left ventricular systolic function during a hospitalization, [29] and are less likely to receive devices like implantable cardioverter-defibrillator (ICD) [30] or undergo coronary revascularization. [31] Whether specific medication prescribing patterns such as hyperpolypharmacy, which is associated with adverse outcomes, can help explain these health disparities is unknown. Indeed, while medication underutilization represents an important form of suboptimal care in HF, medication overutilization may represent an overlooked form of suboptimal care as well. Thus, in light of our finding that low SES is associated with hyperpolypharmacy, there is a need to further explore whether SES-related health disparities in HF can at least in part be explained by hyperpolypharmacy.

There are several strengths to this study. NHANES is a nationally representative sample, and thus has high degree of generalizability to the United States population. Another strength is the detailed list of medications routinely collected from NHANES participants, which are then verified by an in-home visit. There are also some important limitations to our study. This study was observational in nature and thus precluded establishing a causal relationship between variables. Data in NHANES were based on self-report, which can introduce recall bias and social desirability bias. Because details of medication dosing and indications were not available, we were unable to determine whether medications were actually indicated; we also did not have data on the chronicity of medications. Future studies based on medical record abstraction would be useful to better understand this dimension. Also, the number of medications obtained during the in-home visit reflects only prescription medications; accordingly, the prevalence of hyperpolypharmacy may be even greater when accounting for non-prescription medications and dietary supplements. The number of medications did not account for multiple pharmacologically-active ingredients in a single pill (e.g. combination pills), and did not account for pill burden (multiple pills of the same medication); these aspects of medication burden may warrant further investigation. Lastly, details regarding the etiology, subtype (HF with reduced ejection fraction versus HF with preserved ejection fraction), and severity of HF were not available, and could have affected our findings. In particular, given limitations in the sensitivity [32] of self-reported HF, individuals with less severe HF may have been excluded.

## Conclusion

In conclusion, our study showed that hyperpolypharmacy is common in adults with HF and that its prevalence does not vary according to advanced age, functional impairment, or cognitive impairment. Conversely, low household income and low educational status were independently associated with hyperpolypharmacy, even after accounting for influences such as receipt of Medicaid, suggesting that non-medical factors may be contributing to this potentially harmful practice. Future research is warranted to explore the mechanisms underlying this finding and whether hyperpolypharmacy could be contributing to worse health outcomes in this population.

## Abbreviations

HF: Heart failure
NHANES: National Health and Nutrition Examination Survey
SES: Socioeconomic status
